# Manufacturing and Characterization of Hybrid Bulk Voxelated Biomaterials Printed by Digital Anatomy 3D Printing

**DOI:** 10.3390/polym13010123

**Published:** 2020-12-30

**Authors:** Hyeonu Heo, Yuqi Jin, David Yang, Christopher Wier, Aaron Minard, Narendra B. Dahotre, Arup Neogi

**Affiliations:** 1 Department of Physics, University of North Texas, Denton, TX 76203, USA; hyeonu.heo@unt.edu (H.H.); yuqijin@my.unt.edu (Y.J.); 2 Department of Mechanical Engineering, University of North Texas, Denton, TX 76207, USA; 3 Stratasys, Mountain View, CA 94043, USA; David.Yang@stratasys.com (D.Y.); Christopher.Wier@stratasys.com (C.W.); 4 Technical Laboratory Systems, Inc., Katy, TX 77494, USA; Aaron@tech-labs.com; 5 Department of Materials Science and Engineering, University of North Texas, Denton, TX 76207, USA; narendra.dahotre@unt.edu; 6 Center for Agile and Adaptive Additive Manufacturing, University of North Texas, Denton, TX 76207, USA

**Keywords:** digital anatomy 3D printing, voxel, ultrasound elastography, non-destructive testing

## Abstract

The advent of 3D digital printers has led to the evolution of realistic anatomical organ shaped structures that are being currently used as experimental models for rehearsing and preparing complex surgical procedures by clinicians. However, the actual material properties are still far from being ideal, which necessitates the need to develop new materials and processing techniques for the next generation of 3D printers optimized for clinical applications. Recently, the voxelated soft matter technique has been introduced to provide a much broader range of materials and a profile much more like the actual organ that can be designed and fabricated voxel by voxel with high precision. For the practical applications of 3D voxelated materials, it is crucial to develop the novel high precision material manufacturing and characterization technique to control the mechanical properties that can be difficult using the conventional methods due to the complexity and the size of the combination of materials. Here we propose the non-destructive ultrasound effective density and bulk modulus imaging to evaluate 3D voxelated materials printed by J750 Digital Anatomy 3D Printer of Stratasys. Our method provides the design map of voxelated materials and substantially broadens the applications of 3D digital printing in the clinical research area.

## 1. Introduction

The advent of additive manufacturing technology and the improved medical imaging techniques, such as high-resolution CT and MRI scanning, enables the translation of digital images on the computer screens into tangible objects [1,2]. In the biomedical engineering field, additive manufacturing technology can be used for various applications, such as tissue and organ fabrication [3], implant and prostheses production [4], drug delivery [5], and production of anatomical structures [6]. In the clinical research area, especially, 3D-printed anatomical models with realistic anatomical organ shaped structures have been used as experimental models for presurgical planning through rehearsing and preparing complex surgical procedures [7,8,9] to reduce the risk and time on the operating table, as well as education and training [10]. Besides, anatomical 3D printing can demonstrate the performance of the newly developed biomedical products [11]. 

However, several challenges prevent the widespread adoption of anatomical 3D printing. The actual material properties are still far from being ideal, necessitating the need to develop new materials and processing techniques for the next generation of 3D printers optimized for clinical applications. Due to the limitation of traditional 3D printers, the reduction of complex anatomy to a single surface and the simplification of local intensity differences are necessary, resulting in non-natural discontinuities and anatomically inaccurate material properties [1]. To overcome the current issues, recently voxel-based, volumetric, or three-dimensional pixel-based fabrication technique [12] has been introduced, enabling the control of the color or material being used in a design [13]. The voxelated soft matter technique can provide a much broader range of materials and simulate the actual organ that can be designed and fabricated voxel by voxel with high precision.

Despite the utility of voxelated materials, there are a few quantitative comparisons of mechanical properties of the 3D printing materials to the actual organic materials, such as bone [14], heart valves [15], and myocardium [11]. For the practical applications of voxelated 3D printing materials, it is crucial to develop novel high precision material characterization techniques to evaluate the mechanical properties of 3D printed anatomical materials. Currently, it is challenging using conventional methods due to the complexity and the size of the combination of materials.

In mechanical property characterization techniques, the methods can be categorized as destructive and non-destructive techniques. The destructive methods, such as tensile [16] and nanoindentation [17] tests, are usually study-convenient, which can provide elasticity and plasticity from the same samples. On the other hand, non-destructive methods only provide elasticity of the samples. The formal mechanical test is commonly used due to its simplicity. However, that is mainly targeted for hard or homogeneous materials with a large-force delivery load cell so that it is not appropriate for the soft materials like tissue phantoms. Although nano-indentation is more capable of testing biomass such as bone [18], tissue [19], even cells [20], the common nano-indentation platform requires the additional preparation process of specimens and the mounting techniques [21,22], which are not conducive to live tissues or tissue-like phantom used in 3D printed materials. Hence, non-destructive has been considered as a more suitable method for characterizing biomass and tissue phantom.

Ultrasound elastography techniques have been broadly applied in the biomedical field for characterizing the elasticity and its contrast in-vivo [23], the so-called M-mode imaging. Those techniques are also used to characterize the elasticity of soft materials [24]. Conventional elastography techniques utilize ultrasound to measure the deformation induced by external stress or radiational pressure [25]. In the linear elastic deformation range, the externally induced deformation is barely detected by the ultrasound waves. The speed of longitudinal and transversal waves of materials is used to calculate the elasticity [26]. This technique also necessitates the information related to the density and thickness of the tested sample acquired from conventional methods, such as caliper and weight scale [27]. The measurement of density and thickness complex geometries of 3D printed materials are non-trivial and commonly introduces uncertainties.

In 3D printed metals and alloys, dynamic elasticity variations [28] obtained from the measurement of the longitudinal and transversal wave velocity agree well with the static elastic properties measured by mechanical tests. However, due to the dispersion of sound in soft-matter [26], such as hydrogels and composites [29], the difference between static and dynamic moduli can exceed a couple of orders of magnitude [30,31]. The variation in the dynamic elastic modulus of soft-material using elastography measurement can pro-vide useful information about the contrast and the uniformity of 3D printed structures made from soft-material and composite structures.

In this study, to characterize the voxelated 3D printing materials, one layer of Rubik’s cube-like sample was designed with nine different materials, including bone-/tissue-/gel-like materials and mixtures, which are commonly used. That sample was printed by the recently released commercial J750 digital anatomy 3D printer (DAP) of Stratasys. The fabricated materials were characterized by an ultrasonic elastography technique that measures the effective density and the dynamic bulk modulus elastography (EBME) [32,33,34]. Through the EBME technique with the non-destructive and non-invasive measurement setup, effective density and dynamic bulk modulus distribution within the raster-scanned area were calculated.

## 2. Materials and Methods

**Figure 1:**
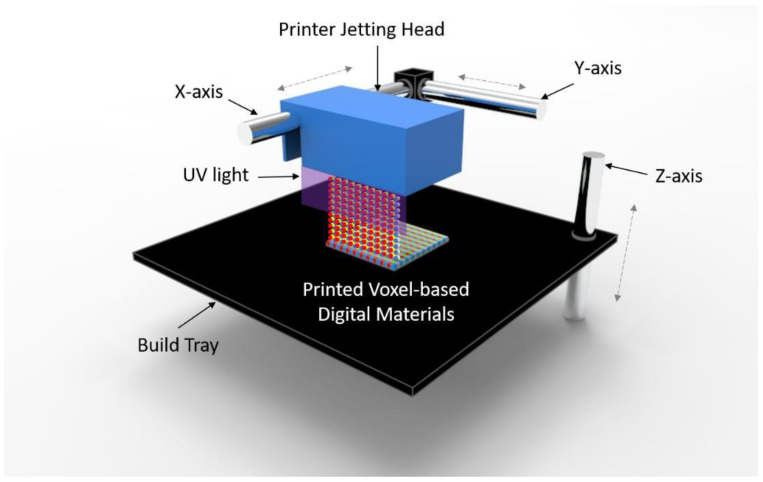
Illustration of Polyjet 3D printing. The printer jetting head moves along the x- and y-axis while printing a digital material on the build tray. The build tray moves up and down along the z-axis during printing. The printed each layer is cured by the UV ramps mounted on the printer head.

Polyjet 3D printing is an additive manufacturing process in which layers of acrylic-based photopolymers are selectively jetted at precise coordinates onto a build tray. Figure 1 is the illustration of polyjet 3D printing. The liquid resin is jet streamed from the print heads via controlled piezoelectric pulses. UV lamps, mounted on the print block, partially cure the resin on each pass. Material is jetted both when the print block travels from left to right and when it travels from right to left. However, when the print block travels from right to left, the Z stage moves upwards slightly, allowing the part to be contacted by the roller mechanism on the print block. The roller, spinning in a clockwise direction, can pick up the partially cured material, thereby leveling the surface of the part for the next printed slice. The roller, on each revolution, is scraped clean by a rollerblade. The tray then moves down again, ready for the next slice to be printed on the next left to right pass. A critical capability of Polyjet technology is the possibility of jetting multiple materials with different properties simultaneously into the same build with micron-level precision. For example, elastomeric materials and rigid materials can be jetted uniformly with a determined ratio, yielding a model with mechanical properties mixed between the two parent materials, called a Digital Material (DM). Similarly, colored resins can be printed to give rise to full-color 3D models closely mimicking traditionally manufactured parts. Optically clear material can also be integrated into builds in this way, allowing for models encased in a “glass” shell, which can prevent more fragile parts from breaking, and creating an aesthetically pleasing model for components such as medical devices or computer chips. As such, Polyjet 3D printing allows for unparalleled design freedom by allowing for manipulation of both mechanical properties and color on a point-by-point basis.

### 2.1. A Voxel-Based Digital Materials (Gel-, Tissue-, and Bone-Like Materials)

**Figure 2:**
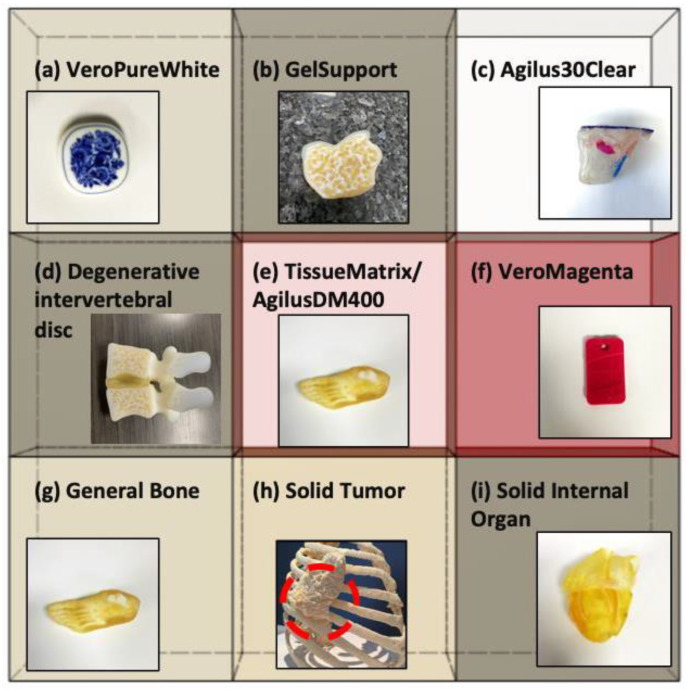
Nine different constitute materials of Rubik’s cube-like matrix sample printed by J750 Digital Anatomy 3D Printer (DAP) of Stratasys using voxelated digital materials (DMs) (Gel-/Tissue-/Bone-like materials) and 3D printed anatomical models printed using each material (inserts). (**a**) VeroPureWhite is a base material and has a white color. (**b**) GelSupport is utilized for printing small blood vessels or porous space in bones. (**c**) Agilus30Clear is a transparent base material. (**d**) A DM to represent a degenerative intervertebral disc that is slightly dense. (**e**) TissueMatrix/AgilusDM400, 400 refers to 400 μm agilus “skin,” representing soft anatomy, commonly things like muscle, fat, and skin. (**f**) VeroMagenta is a base material having magenta color. (**g**) General bone represents any bone that is non-vertebrae, skull, long bone, or ribs. (**h**) A DM to represent a tumor in the bone. (**i**) A DM to represent a solid internal organ, any solid internal organ. Tech- Labs and Stratasys took the pictures of all anatomical models.

The Digital Anatomy Printer solution by Stratasys is a combination of unique, innovative printing materials and proprietary software. The novel printing materials include GelMatrix, TissueMatrix, and BoneMatrix, all of which are printed as DMs. Figure 2 shows the 3D-printed sample having Rubik’s cube-like matrix shape consisting of nine different digital materials to be utilized for the material characterization using the EBME technique. Moreover, the example of 3D printed anatomical models by each material is inserted in the matrix to show the actual applications of DMs. GelMatrix is an alkaline solution soluble material utilized when printing small features and, most importantly, in blood vessel type structures. The GelMatrix material is softer and easier to remove and dissolve than standard Support706B, utilized in other Stratasys Polyjet printing platforms. It allows for realizing a small diameter of blood vessels like structure previously unattainable by 3D printing. Due to its softness, GelMatrix is printed in conjunction with normal Support706B material and Agilus30Clear, to give models enough support during the printing process. The TissueMatrix material is a soft material utilized to mimic tissue anatomy. This material has a shore value of 30 on the Shore00 scale, like the feeling of a gummy bear or a gel shoe insert, which is the softest 3D printable material in the market today. TissueMatrix is typically printed with Agilus30Clear for handling, due to the propensity of TissueMatrix being rather adhesive. BoneMatrix material is a high toughness material printed to simulate bone models. The material has excellent shape memory properties, able to be bent and still retain its original shape. It also has superior mechanical properties compared to prior materials. BoneMatrix is printed in conjunction with VeroPureWhite, which gives bone models their characteristic white color.

The DAP software is employed to stream each material to a specific location in the matrix. It is a unique class of the Polyjet family that implements the voxel level control into models and provide material architectures that mimic human-like anatomical tissues. Contrary to the standard Polyjet jet technique, which uniformly mixes materials to provide a specific material property or color, the DAP software dynamically mixes the materials based on the geometry. For example, when printing a femur, there is a cortical bone layer on the exterior and a cancellous bone layer in the interior. The DAP software has several presets, capable of mimicking dense bone to porous bone, which dynamically changes the model’s architecture with just a click. The software can also change the layer thickness based on the model’s overall geometry and user inputs, i.e., smaller femur bones will have proportionally thinner cortical layers than larger ones. There are hundreds of presets in the software that allows the user to manipulate models to simulate human tissue.

### 2.2. Experiment Setup

**Figure 3:**
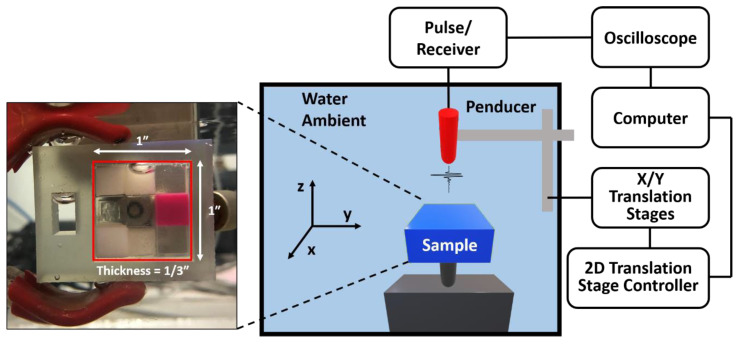
The schematic of the experimental setup of the dynamic bulk modulus elastography (EBME) raster scan and the dimension of the printed sample. The sample has Rubik’s cube-like shape with 9 different materials. The sample was printed by J750 DAP of Stratasys.

The dimension of the sample and the schematic diagram of the experimental setup is shown in Figure 3. The sample was designed as one layer of a Rubik’s cube-like shape with nine different materials. Each material is the 1/3 × 1/3 × 1/3 cubic inch-material was printed by the J750 Digital Anatomy 3D Printer (Stratasys, Eden Prairie, MN, USA). We scanned a 20 mm × 20 mm area on the 3D printed sample block using the EBME technique underwater. An Olympus Panametrics V316-N-SU (Olympus-IMS, Waltham, MA, USA) 0.125-inch diameter 20 MHz unfocused immersion transducer was used to generate a pulse, 10–35 MHz with a repetition rate of 200, for the raster scan and record signals reflected by the samples. A JSR Ultrasonic DPR 300 Pulse/Receiver (Imaginant, Inc., Pittsford, NY, USA) internally operated the pulse source and a time trigger. The data were collected by a Tektronix MDO 3024b (Tektronics Inc., Beaverton, OR, USA). For the raster scan, the three axes translation stages controlled by the three axes motion controller, LC Series Linear Stages of Newmark Systems, Inc., was used. The scanned area of the sample was 20 mm × 20 mm at 0.5 mm interval alongs the x- and y-axis for both. The acquisition rate was 512 signals per 20 s.

## 3. Theoretical Framework

The effective dynamic bulk modulus and density can be determined by the acoustic impedance and the longitudinal speed of sound of the measured sample through their relation. Here, we summarized the mechanism of EBME with equations to provide the acoustic impedance of samples. This mechanism is based on the analysis of the reflected short acoustic pulses, i.e., echos, from the boundary of samples that are embedded in the fluid with the known mechanical properties. Then, the unknown impedance of the sample can be obtained through the relation between the input signal, the first reflected signal, and the second reflected signal. The detailed mathematical works are provided in the sup-plementary materials. 

The sample acoustic impedance in the raster-scanned imaging can be written as [32],

(1) $$Z_{1}=\;Z_{0}\left(\frac{-1-\frac{p_{1}}{p_{e}-p_{0}}-\sqrt{4\frac{p_{1}}{p_{e}-p_{0}}+1}}{\frac{p_{1}}{p_{e}-p_{0}}-2}\right)\;,\;\frac{Z_{1}}{Z_{0}}\geq 1,\;Z_{1}=\;Z_{0}\left(\frac{\left(1-\frac{p_{1}}{p_{e}-p_{0}}\right)+\sqrt{1-4\frac{p_{1}}{p_{e}-p_{0}}}}{\frac{p_{1}}{p_{e}-p_{0}}+2}\right)\;,\;\frac{1}{3}\leq \frac{Z_{1}}{Z_{0}}<1.$$

where 𝑝_e_ is the pressure amplitude of the emission pulse from the ultrasound penducer, 𝑝_0_ is the pressure amplitude of the first echo that occurred from the first interface between water ambient and the measured sample, and 𝑝_1_ is the pressure amplitude of the reflection from the second boundary between the sample and water ambient. 𝑝_e_ is measured from a separate bistatic calibration without any sample in the ambient water. 𝑝_0_ and 𝑝_1_ are obtained from the raster scan imaging. 𝑐 is the sample sound velocity obtained from the time of flight of the wave in the tested sample at the measured position during the scan, described as 𝑐 = 2𝑑/(𝑇_1_ − 𝑇_0_), where 𝑇_1_ and 𝑇_0_ are the first peaks of the first and second reflected signals are selected from the absolute maximum values of each pulse (see Supplementary Materials). 𝑑 is the sample thickness. *Z*_1_ is the impedance of the tested sample at the scanned position. The ambient water impedance is known and defined as *Z*_0_ = 𝜌_0_*c*_0_, where 𝜌_0_ = 1000 kg/m^3^ and 𝑐_0_ = 1480 m/s at room temperature.

In this study, the time point of starting the pulse is important to evaluate the time delay and calculate the sound velocity values. The MATLAB^®^ pre-programmed peak finding function was applied to localize the positive peaks and negative valleys. The time point and amplitude of the first and second peaks or valleys were the 𝑝_0_ and 𝑝_1_ in the calculation. During the calibration for determining 𝑝_e_ the time window was moved to the center of the received signal 𝑝_e_ with the sample rate as the EBEM scan. The longitudinal sound velocity *c* in the sample was calculated by the time delay between two measured reflected signals obtained as reflections from its boundaries. From impedance and speed of sound, the bulk modulus (Equation (2)) and mass density (Equation (3)) of each elastic layer are easily calculated as

𝐾_𝑑𝑦𝑛_ = 𝜌𝑐^2^ = 𝑍𝑐, (2)

(3) $$\rho_{eff}=\frac{Z}{C}.$$

## 4. Results

**Figure 4:**
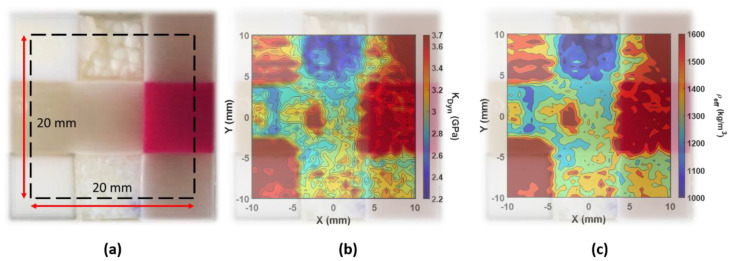
(**a**) The photograph of the 3D printed sample consisting of nine different materials described in Figure 2. The scanned area is depicted with the dashed line (20 mm × 20 mm). (**b**) The dynamic bulk modulus elastography, ranging from 2.2 GPa to 3.7 GPa, overlapped with the photograph of the sample. (**c**) The effective density distribution map, ranging from 1176 kg/m^3^ to 1625 kg/m^3^, overlapped with the photograph of the sample.

The EBME scan, the elasticity distribution, was shown in Figure 4. The dynamic bulk modulus map and the effective density map illustrated a well fused nine materials combination. With the photograph overlapped with the scanned results, the smooth boundaries between the materials cannot be clearly identified because of the connective interfaces. The region of softer materials attached to hard materials provides a smaller deformation response due to the stress and results in a higher estimate of the elasticity that is measured.

Figure 4b shows the EBME scanned dynamic bulk modulus distribution of nine different materials. The color scale was in the range between 2.2 GPa to 3.7 GPa, where the typical bulk modulus of DI-water is about 2.1 GPa which is not frequency-dependent in the given ultrasound range. In the tested sample, the soft tissue materials (upper-center/-right, middle-center/-left, and lower-center/-right) had lower dynamic bulk modulus values, below 3.2 GPa. Among them, the lower-center (tumor phantom) provided a higher averaged dynamic modulus. In the phantom tumor area, the microstructure was formed where softer tissue phantom is embedded in the small harder tumor phantom pullets. The EBME scan also illustrated the interval structure of the tumor pullets in the dynamic modulus map. Besides those softer phantoms, the other three materials depict a significantly higher dynamic bulk modulus (upper-left, lower-left, and middle-right), as expected. Those materials are the phantoms for harder anatomy such as bone, which had high dynamic elasticity beyond 3.2 GPa.

In Figure 4c, the EBME scanned effective density map is exhibited. The effective density variation range in the scanned map was from 1176 kg/m^3^ to 1625 kg/m^3^. The general property difference in the effective density map was well-matched with the dynamic bulk modulus map shown in Figure 4b. Besides the upper center attenuated material region, the other materials have an effective density at 20 MHz, all above 1150 kg/m^3^. Due to the dehydration and rehydration, those two regions of the materials, upper- and lower-center, had thinner thickness values comparing with other materials, which introduced uncertainty in the EBME measurements. The contrasting behavior can be indicative of the non-uniform thickness in the dynamic bulk modulus and effective density maps.

The results are summarized in Table 1 and compared with the reference values, such as elastic modulus and polymerized density, given by the provider, Stratasys [35]. Unfor-tunately, there is no such material property for the digital mixtures because it is too soft to measure elasticity using the conventional mechanical testing methods for the gel-like material. Moreover, the material properties of digital materials are defined case by case, and they would be fully anisotropic. Even a material itself is not ho-mogeneous as shown in Figure 4.

For the given properties, by assuming Poisson’s ratio, about 3.5–4, the static bulk modulus of (a) VeroPureWhite and (f) VeroMagenta can be calculated, and that value is similar to the dynamic bulk modulus measured by the EBME technique. However, the effective density is quite different, about 24–37%, with the reference value, polymerized density. It is because the effective values are influenced by the correlation between the size of microstructure, approximately 14–27 μm, and the wavelength of 20 MHz, 75 μm.

## 5. Discussion

In practical biomedical imaging applications, phased array transducers are commonly used [24]. These normally yield much lower resolution and worse signal- to-noise ratio than immersion plane wave transducers or immersion focusing transducers without advanced signal processing procedures. The proposed material characterization techniques can distinguish material properties, effective bulk modulus, and effective dynamic density, with high resolution, about ±4%. Hence, this technique is suitable to characterize material properties of a sample printed by additive manufacturing which has anisotropic properties of inhomogeneous materials. This study focused on bio-printed objects for proving the concept of inspecting materials and interfaces. However, in principle, this technique could be applied to other manufacturing processes and other 3D printing techniques, such as casting, molding, and other additive manufacturing, e.g., FDM and SLM. In any manufacturing process where the porosity or density affects the quality of the manufactured, the processing quality may be monitored or inspected by comparing effective density measurements to a standard calibrated reference. Increased resolution may be addressed by implementing an acoustic lens [36] to narrow the wave-sample interaction region or increase signal- to-noise ratios.

## 6. Conclusions

In this study, we used the recently developed ultrasound elastography technique to characterize voxelated materials printed by J750 DAP, polyjet type 3D printer, which can print tissue-/bone-/gel-like materials and mixtures. This printing technique has unparalleled design freedom by manipulating both mechanical properties and color on a voxel-by-voxel basis. Therefore, the high precision material characterization technique (Spatial resolution and magnitude) is critical to differentiate the distribution of material properties. Our method demonstrated the capability to provide the design map of voxelated materials and substantially broaden the applications of 3D digital printing in the clinical research area. Furthermore, this technique can be used as a health monitoring system of the 3D printer itself because the printer has aging effects through the continuing operation resulting in incomplete printing or defects and voids in the sample.

## Figures and Tables

**Table 1:** Summary of the comparison of material properties between EBME results, such as dynamic bulk modulus, effective density and average speed of sound, and reference values, such as elastic modulus and polymerized density, given by the provider, Stratasys [35].

Material	Dynamic Bulk Modulus, *K_dyn_* (GPa)	Reference Modulus of Elasticity, *Eref_static* (GPa) [35]	Effective Density, *Ρ_eff_* (kg/m^3^)	Reference Polymerized Density, *Ρ_ref_* (kg/m^3^) [35]	Average Speed of Sound (m/s)
(a) VeroPureWhite	3.35 ± 0.13	2.0–3.0	1452 ± 70	1170–1180	1590 ± 40
(b) GelSupport	2.55 ± 0.09	N/A	1176 ± 40	N/A	1625 ±18
(c) Agilus30Clear	3.47 ± 0.17	N/A	1532 ± 66	1140–1150	1478 ± 26
(d) Degenerative intervertebral disc	2.99 ± 0.16	N/A	1274 ± 80	N/A	1642 ± 54
(e) TissueMatrix/AgilusDM400	3.08 ± 0.14	N/A	1342 ± 75	N/A	1600 ± 38
(f) VeroMagenta	3.69 ± 0.06	2.0–3.0	1609 ± 33	1170–1180	1425 ± 15
(g) General Bone	3.62 ± 0.05	N/A	1625 ± 30	N/A	1370 ± 11
(h) Solid Tumor	3.26 ± 0.12	N/A	1412 ± 61	N/A	1635 ± 29
(i) Solid internal organ	3.25 ± 0.12	N/A	1403 ± 63	N/A	1651 ± 37

## Data Availability

The data presented in this study are available on request from the corresponding author.

## Supplementary Materials

The following are available online at https://www.mdpi.com/2073-4360/13/1/123/s1, Figure S1: Numerical simulation of EBME test on a single measurement of a bone-like material in ambient DI water.

## References

[1] Hosny A., Keating S.J., Dilley J.D., Ripley B., Kelil T., Pieper S., Kolb D., Bader C., Pobloth A.M., Griffin M. (2018). From Improved Diagnostics to Presurgical Planning: High-Resolution Functionally Graded Multimaterial 3D Printing of Biomedical Tomographic Data Sets. 3D Print. Addtive Manuf..

[2] Marro A., Bandukwala T., Mak W. (2016). Three-dimensional printing and medical imaging: A review of the methods and applications. Curr. Probl. Diagn. Radiol..

[3] Murphy S.V., Atala A. (2014). 3D bioprinting of tissues and organs. Nat. Biotechnol..

[4] Mavroidis C., Ranky R.G., Sivak M.L., Patritti B.L., DiPisa J., Caddle A., Gilhooly K., Govoni L., Sivak S., Lancia M. (2011). Patient specific ankle-foot orthoses using rapid prototyping. J. Neuroeng. Rehabil..

[5] Alhnan M.A., Okwuosa T.C., Sadia M., Wan K.W., Ahmed W., Arafat B. (2016). Emergenceof3D printed dosage forms: Opportunities and challenges. Pharm. Res..

[6] McGurk M., Amis A.A., Potamianos P., Goodger N.M. (1997). Rapid prototyping techniques for anatomical modelling in medicine. Ann. R. Coll. Surg. Engl..

[7] Cui J., Chen L., Guan X., Ye L., Wang H., Liu L. (2014). Surgical planning, three-dimensional model surgery and preshaped implants in treatment of bilateral craniomaxillofacial post-traumatic deformities. J. Oral Maxillofac. Surg..

[8] Jacobs S., Grunert R., Mohr F.W., Falk V. (2008). 3D-Imaging of cardiac structures using 3D heart models for planning in heart surgery: A preliminary study. Interact. Cardiovasc. Thorac. Surg..

[9] Hosny A., Shen T., Kuo A.S., Long D., Andrawes M.N., Dilley J.D. (2018). Unlocking vendor-specific tags: Three-dimensional printing of echocardiographic data sets. J. Thorac. Cardiovasc. Surg..

[10] Kiarashi N., Nolte A.C., Sturgeon G.M., Segars W.P., Ghate S.V., Nolte L.W., Samei E., Lo J.Y. (2015). Development of realistic physical breast phantoms matched to virtual breast phantoms based on human subject data. Med. Phys..

[11] Severseike L., Lee V., Bakken C., Brandon T., Bhatia V. (2019). Polyjet 3D printing of tissue-mimicking materials: How well can 3D printed synthetic myocardium replicate mechanical properties of organic myocardium?. BioRxiv.

[12] Doubrovski E.L., Tsai E.Y., Dikovsky D., Geraedts J.M., Herr H., Oxman N. (2015). Voxel-based fabrication through material property mapping: A design method for bitmap printing. Comput. Aided Des..

[13] Erickson A. Create the Future with New Grabcad Voxel Print. https://www.cati.com/blog/2017/12/create-the-future-with-new-grabcad-voxel-print/.

[14] Wood Z., Lynn L., Nguyen J.T., Black M.A., Patel M., Barak M.M. (2019). Are we crying Wolff? 3D printed replicas of trabecular bone structure demonstrate higher stiffness and strength during off-axis loading. Bone.

[15] Vulkicevic M., Puperi D.S., Grande-Allen K.J., Little S.H. (2017). 3D Printed Modeling of the Mitral Valve for Catheter-Based Structural Interventions. Ann. Biomed. Eng..

[16] Masood S.H., Mau K., Song W.Q. (2010). Tensile properties of processed FDM polycarbonate material. Mater. Sci. Forum.

[17] Calabri L., Pugno N., Menozzi C., Valeri S. (2008). AFM nanoindentation: Tip shape and tip radius of curvature effect on the hardness measurement. J. Phys. Condens. Matter.

[18] Hengsberger S., Kulik A., Zysset P.H. (2002). Nanoindentation discriminates the elastic properties of individual human bone lamellae under dry and physiological conditions. Bone.

[19] Sun J.-y., Tong J. (2007). Fracture toughness properties of three different biomaterials measured by nanoindentation. J. Bionic Eng..

[20] Gindl W., Gupta H.S. (2002). Cell-wall hardness and Young’s modulus of melamine-modified spruce wood by nano-indentation. Compos. Part A Appl. Sci. Manuf..

[21] Ramesh Babu S., Jaskari M., Järvenpää A., Porter D. (2019). The effect of hot-mounting on the microstructure of an As-Quenched auto-tempered low-carbon martensitic steel. Metals.

[22] Chusuei C.C., Gwynn L.K., Schreifels J.A. (1992). Two-level liquid-nitrogen-cooled sample mounting assembly for surface analysis. Rev. Sci. Instrum..

[23] Islam M.T., Chaudhry A., Tang S., Tasciotti E., Righetti R. (2018). A new method for estimating the effective Poisson’s ratio in ultrasound poroelastography. IEEE Trans. Med. Imaging.

[24] Nightingale K. (2011). Acoustic radiation force impulse (ARFI) imaging: A review. Curr. Med. Imaging Rev..

[25] Gennisson J.-L., Deffieux T., Fink M., Tanter M. (2013). Ultrasound elastography: Principles and techniques. Diagn. Interv. Imaging.

[26] Majumdar P., Singh S.B., Chakraborty M. (2008). Elastic modulus of biomedical titanium alloys by nano-indentation and ultrasonic techniques—A comparative study. Mater. Sci. Eng. A.

[27] Kupperman D.S., Reimann K.J., Abrego-Lopez J. (1987). Ultrasonic NDE of cast stainless steel. NDT Int..

[28] Wang T., Shukla S., Nene S.S., Frank M., Wheeler R.W., Mishra R.S. (2018). Towards Obtaining Sound Butt Joint Between Metallurgically Immiscible Pure Cu and Stainless Steel Through Friction Stir Welding. Metall. Mater. Trans. A.

[29] Jin Y., Heo H., Walker E., Krokhin A., Choi T.Y., Neogi A. (2020). The effects of temperature and frequency dispersion on sound speed in bulk poly (Vinyl Alcohol) poly (N-isopropylacrylamide) hydrogels caused by the phase transition. Ultrasonics.

[30] Walker J.M., Myers A.M., Schluchter M.D., Goldberg V.M., Caplan A.I., Berilla J.A., Mansour J.M., Welter J.F. (2011). Nondestructive evaluation of hydrogel mechanical properties using ultrasound. Ann. Biomed. Eng..

[31] Jin Y., Yang T., Ju S., Zhang H., Choi T.-Y., Neogi A. (2020). Thermally Tunable Dynamic and Static Elastic Properties of Hydrogel Due to Volumetric Phase Transition. Polymers.

[32] Jin Y., Walker E., Krokhin A., Heo H., Choi T.-Y., Neogi A. (2019). Enhanced instantaneous elastography in tissues and hard materials using bulk modulus and density determined without externally applied material deformation. IEEE Trans. Ultrason. Ferroelectr. Freq. Control.

[33] Jin Y., Walker E., Heo H., Krokhin A., Choi T.-Y., Neogi A. (2020). Nondestructive ultrasonic evaluation of fused deposition modeling based additively manufactured 3D-printed structures. Smart Mater. Struct..

[34] Jin Y., Yang T., Heo H., Krokhin A., Shi S.Q., Dahotre N., Choi T.Y., Neogi A. (2020). Novel 2D Dynamic Elasticity Maps for Inspection of Anisotropic Properties in Fused Deposition Modeling Objects. Polymers.

[35] PolyJet 3D Printers Systems and Materials-Stratasys. https://www.stratasys.com/-/media/files/printer-spec-sheets/polyjet-systems-and-materials-overview-en-a4.pdf.

[36] Walker E.L., Reyes-Contreras D., Jin Y., Neogi A. (2019). Tunable Hybrid Phononic Crystal Lens Using Thermo- Acoustic Polymers. ACS Omega.

