# Supplementary material for: Manufacturing and Characterization of Hybrid Bulk Voxelated Biomaterials Printed by Digital Anatomy 3D Printing
Source: Polymers (Basel). 2020 Dec 30;13(1):123. doi: 10.3390/polym13010123 (PMC7796254; doi:10.3390/polym13010123)
Supplement: Supplementary file 1 [file polymers-13-00123-s001.pdf]

Manufacturing and Characterization of Hybrid Bulk Voxelated Biomaterials Printed by Digital Anatomy 3D Printing

Hyeonu Heo [1], Yuqi Jin [1,2], David Yang [3], Christopher Wier [3], Aaron Minard [4], Narendra B. Dahotre [5,6], and Arup Neogi [1,6,*]

[1] Department of Physics, University of North Texas, Denton, TX 76203, USA; hyeonu.heo@unt.edu (H.H.); yuqijin@my.unt.edu (Y.J.); arup.neogi (A.N.)
[2] Department of Mechanical and Energy Engineering, University of North Texas, Denton, Texas 76207, USA;
[3] Stratasys, Mountain View, CA 94043, USA; David.Yang@stratasys.com (D.Y.); Christopher.Wier@stratasys.com (C.W.)
[4] Technical Laboratory Systems, Inc., Katy, TX 77494, USA; Aaron@tech-labs.com (A.M.)
[5] Department of Materials Science and Engineering, University of North Texas, Denton, Texas 76207, USA; narendra.dahotre@unt.edu (N.D.)
[6] Center for Agile and Adaptive Additive Manufacturing, University of North Texas, Denton, Texas 76207, USA
* Correspondence: Arup Neogi: arup.neogi@unt.edu

EBME Theory [1]

In fluid medium, the velocity is the potential field as $\vec{V} = \nabla\varphi$ described. When the fluid is homogeneous, the potential φ is the factor of standard wave equation, $c^{-2}\ddot{\varphi} + \nabla^2\varphi = 0$, with sound velocity c. The oscillation of the pressure wave can also represent by the potential as $p = -\rho\dot{\varphi}$, where ρ is the density of fluid medium. Since the operation of effective dynamic density and bulk modulus elastography (EBME) using the short pulse envelope instead of continuous wave, the reflection of the pulse is different from the CW such as a sine wave.

A short pulse $\varphi_e(T)$ is emitted by a transducer from a flat object, where T is the time factor. The entire package of energy separates into two parts. The first part is the reflected echo from the closer boundary of the sample named as $\varphi_0(T)$. The second one is transmitted into the target object. The temporal width of the original pulse $\varphi_e(T)$ has to be short enough comparing with the target sample thickness. Due to this condition, the first boundary maintains stable when the roundtrip pulse arrives there. In another words, the duration of the pulse is needed to be long enough to eliminate the frequency dependent dispersion on the acoustic impedance values of ambient fluid Z_0 and the target sample Z_1. The linear relation in amplitude and length between the input and output pulses on the targe sample can be represented by single frequency component planar waves with angular frequency ω and its wave vector as $k_0 = \omega/c_0$ and $k_1 = \omega/c_1$ stated.

The dependence between the source pulse φ_e, the first echo φ_0, and the transmitted energy $\varphi_t$is occurred at the interface between the ambient fluid and a sample, where the acoustic pressure and velocity of the wave are linear and continuous as $p_e + p_0 = p_t$ and $V_e + V_0 = V_t$. In the potential form of wave equation, then the source and two separated pulses are written as $\varphi_e(x,T) = e^{ik_1x-i\omega t}$, $\varphi_0(x,T) = r_{0,1}e^{-ik_0x-i\omega t}$, $\varphi_t(x,T) = t_{0,1}e^{ik_1x-i\omega t}$ where $r_{0,1}$ and $t_{0,1}$ are the reflection and transmission coefficients expressed as $t_{0,1} = 2Z_1/(Z_0 + Z_1)$ and $r_{0,1} = \frac{Z_1-Z_0}{Z_1+Z_0}$. The, the wave velocity is converted in terms of pressure as $p_t = \frac{Z_1}{Z_0}(p_e - |p_0|)$ when $Z_1 > Z_0$ and $p_t = \frac{Z_1}{Z_0}(p_e + |p_0|)$ when $Z_1 < Z_0$. In the experiment, the signal of p_0 can be found by comparing with a known impedance hard object in the same

ambient fluid, or only acquired the absolute amplitude of the echo without any comparison. In the second case, the impedance of the sample Z_1 can be estimated with the recorded amplitude of the second reflected signal p_1. Transmission of the internal roundtrip signal through the front boundary of the sample separated its energy left in the sample, the relation the roundtrip echo and front surface echo was expressed in terms of the emission source amplitude as $p_1 = t_{1,0}\, r_{1,0}\, p_t = \frac{2Z_0|Z_1-Z_0|}{(Z_1+Z_0)^2}[p_e - \mathrm{sgn}(Z_1 - Z_0)|p_0|]$.

In experiments, all three signals, p_e, $|p_0|$, and p_1 can be measured and combined into constant as $\alpha = \frac{p_1}{p_e - \mathrm{sign}(Z_1-Z_0)|p_0|}$. The sample impedance Z_1 can be calculated based on this experimentally measured constant α as follows $\frac{Z_1}{Z_0} = \frac{-1-\alpha-\sqrt{4\alpha+1}}{\alpha-2}$ when $\frac{Z_1}{Z_0} \geq 1$, $\frac{Z_1}{Z_0} = \frac{(1-\alpha)+\sqrt{1-4\alpha}}{\alpha+2}$ in the range of $\frac{1}{3} < \frac{Z_1}{Z_0} < 1$, $\frac{Z_1}{Z_0} = \frac{(1-\alpha)-\sqrt{1-4\alpha}}{\alpha+2}$ when $0 < \frac{Z_1}{Z_0} \leq \frac{1}{3}$. In the experimental data, $|p_0|$ and p_1 are taken from the corresponding absolute maximum values of the measured first and second reflected pulses. The source amplitude p_e is the absolute maximum values of the transducer emission in the ambient medium without any sample using bistatic setup measurement. The speed of sound value c in the target sample can be determined from the time delay between two reflected echo which the known sample thickness value. Based on the estimated sample impedance and speed of sound, the dynamic bulk modulus and effective density of the scanned sample can be calculated as $K = Zc$ and $\rho = \frac{Z}{c}$.

Numerical Simulation

For a better view of the EBME measurement, we perform two numerical simulation which provides the propagation of pulse interacting with sample being tested (see Figure S1). In the first simulation, we place a bone-like material with higher physical property comparing with ambient water. A transducer is located at the top of the DI water tank. The target sample is placed at the middle region of the water tank. In Figure S1 (A), we collect two reflected envelopes p_0 and p_1 from the front and back surface of the sample due to clear impedance mismatch between the ambient water and the target sample.

In the insets, the simulated sound pressure field is plotted in the entire study area at three time points. From left to right, the first inset shows a pulse is excited from the transducer. The second inset shows the pulse travels at the back boundary of the scanned sample. On the upper side of this inset, a reflected pulse is on the way approaching back to the transducer which is the first echo p_0 occurred from the front interface between ambient water and sample. In the third inset, along the continuous moving of the time, the main pulse travels to a lower position which completely passes the sample, and another reflection moving backward to the transducer occurs from the back boundary of the sample.

In Figure S1 (B), the simulation is duplicated bistatic setup calibration for obtaining the value of transducer emits source pulse amplitude p_e. In the insets, the numerical experimental setup does not involve sample. The pulse envelope transmits through low attenuation and non-dispersion DI water ambient arriving at another receiver transducer. From the blue line in (A) and red line in (B), the relative amplitude relation between the p_e, p_0 and p_1 can be comparing and visualized. The typical values of p_e, p_0 and p_1 are selected from the absolute maximum values of each pulses. For the source pulse in (B), p_e value can be obtained based on the amplitude of the second positive peak. In (A), the p_0 and p_1 are able to be found from the second positive peak of the first echo and the second negative peak on the second echo.

References

1. Jin, Y.; Walker, E.; Krokhin, A.; Heo, H.; Choi, T. Y.; Neogi, A. Enhanced instantaneous elastography in tisseus and hard materials using bulk modulus and density determined without externally applied

material deformation. *IEEE Transactions on Ultrasonics, Ferroelectrics, and Frequency Control* **2019,** *67* (3), 624-634.

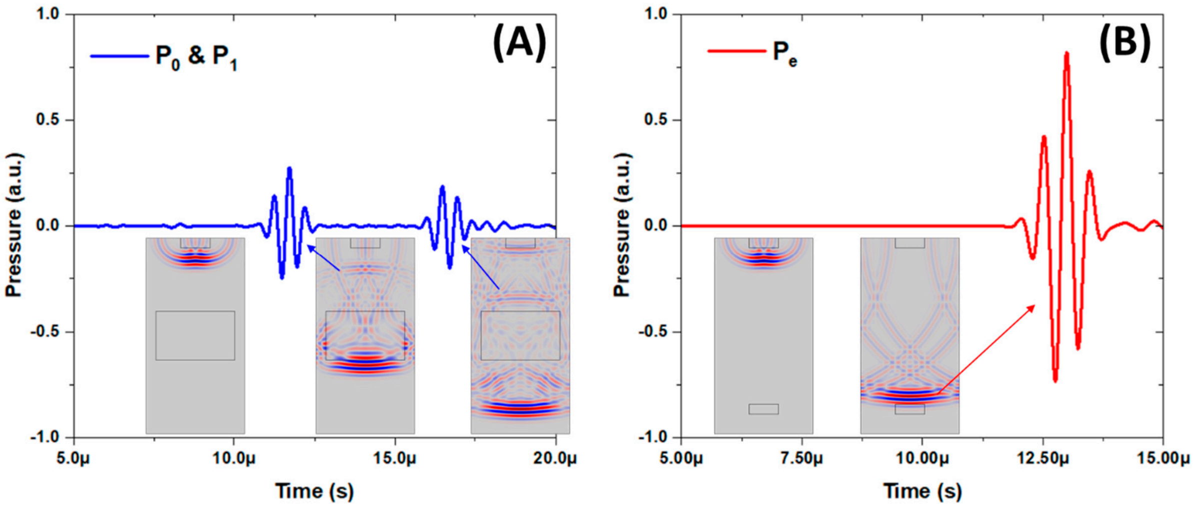

Figure. S1. Numerical simulation of EBME test on a single measurement of a bone-like material in ambient DI water. (**A**) Two reflected echoes from the front and back boundary of the tested sample $p_0$and p_1. (**B**) The bistatic calibration of emission source amplitude p_e in water ambient without sample.
